# Evaluation of a Nepalese-Language Version of the Oral Health Impact Profile Scale Applied to Periodontal Disease (OHIP-14-PD): A Reliability and Validation Study

**DOI:** 10.1155/ijod/7031395

**Published:** 2025-10-14

**Authors:** Arjun Hari Rijal, Pratistha Ghimire, Simant Lamichhane, Sagar Adhikari, Manoj Humagain

**Affiliations:** ^1^Department of Periodontology and Oral Implantology, Kathmandu University School of Medical Sciences, Dhulikhel, Kavrepalanchok, Nepal; ^2^Dental Department, Methinkot Hospital, Bhakundebesi, Kavrepalanchok, Nepal; ^3^Department of Oral Medicine and Maxillofacial Radiology, Kathmandu University School of Medical Sciences, Dhulikhel, Kavrepalanchok, Nepal

**Keywords:** chronic periodontal disease, oral health, oral health-related quality of life, oral hygiene status

## Abstract

**Background:**

Chronic periodontal disease (PD) is a significant oral health issue affecting ~18% of the adult population in Nepal. While there have been advancements in understanding its causes, prevention, and management, the occurrence and disease severity have not significantly decreased This study aims to adapt and validate the oral health impact profile applied to PD (OHIP-14-PD) questionnaire by experts to evaluate the impact of PD on oral health-related quality of life (OHRQoL) in the Nepalese population using the Nepalese language.

**Methods:**

After completion of translation, validation, and cross-cultural adaptation process in 85 patients with chronic PD, the internal consistency of the questionnaire was calculated using Cronbach's alpha value. Spearman's correlation coefficient was used to determine the validity. Similarly, Cohen's kappa values were used to find out the agreement among the responses in the test–retest process. The intraclass correlation coefficient (ICC) was used to find out the consistency of the questionnaire with a 95% confidence interval.

**Results:**

The internal consistency was found to be good for the translated and validated questionnaire to use in the Nepali hospital (Cronbach's alpha value = 0.868, range = 0.85–0.86). The pre- and post-test Cohen's kappa value showed good to almost perfect consistency of agreement (Cohen's kappa value = 0.788–0.958). Spearman's correlation coefficient for the total OHIP-14-PD Nepali version was 0.585.

**Conclusion:**

The Nepali language version OHIP-14-PD is a reliable, content/construct validated tool which can be used to find out the impact of PD on OHRQoL.

## 1. Background

Chronic periodontal disease (PD) is a significant oral health issue affecting ~18% of the adult population in Nepal [1]. While there have been advancements in understanding its causes, prevention, and management, the occurrence and disease severity have not significantly decreased [2]. Clinicians often evaluate the seriousness of PD using clinical measures such as bleeding on probing, pocket depth, and clinical attachment level. However, symptoms like redness, bleeding, tooth mobility, and bad breath, which are important to patients and greatly affect their quality of life (QoL), are often not recorded in research studies [3]. PD can also affect various functional aspects, including chewing, swallowing, speech, and esthetics, thereby influencing the self-esteem of an individual [4]. Individuals with periodontitis generally experience a lower QoL compared to those with healthy gums [3, 5]. It's important to understand how individuals view their oral health and its significance, as these perceptions affect their willingness to seek the right treatment [5]. More and more researchers are becoming interested in the idea of “QoL” and its effects on oral health-related QoL (OHRQoL) [6].

The effects of chronic PD on OHRQoL can be evaluated using various measures [7–9]. The most commonly used tool for the measurement of OHRQoL is Oral Health Impact Profile (OHIP) [10]. It usually measures the effects of any oral diseases with the help of the following parameters: “physical pain, physical disability, psychological discomfort, psychological disability, functional limitation, social disability, and handicap.” It includes 49 questions to find out the effects of oral diseases on QoL, which is difficult to use for the regular research process. To overcome this, a shorter version of the OHIP was created, that is, OHIP-14 [11]. However, OHIP-14 is a generalized questionnaire that assesses the overall effect of oral conditions on QoL. Therefore, there is a need for a scale that can measure the effects of PD on OHRQoL.

One such measure is the OHIP to periodontal disease (OHIP-14-PD) [12], which is a well-validated tool for assessing OHRQoL. It consists of 14 questions similar to OHIP-14, but is applicable to evaluate patients with PDs. It measures the effects of PDs on OHRQoL using six dimensions as in the original OHIP-14. This tool is especially helpful for predicting overall mental well-being and life satisfaction, and researchers have tested how well it can detect changes over time. This tool allows for the evaluation of the effects of chronic PD and can detect variations in the QoL of patients pre- and post-treatment. OHIP-14-PD is known for its good internal consistency reliability, attributed to its higher number of items compared to other OHRQoL measures. There are reports of using OHIP-14-PD in the Mexican population [12, 13], while there is a lack of a Nepalese version of OHIP-14-PD. Therefore, this study aimed to translate the OHIP-14-PD into the Nepalese language and find out its reliability, validity, and cross-cultural adaptation in patients with PD.

## 2. Methods

### 2.1. Participants

A descriptive cross-sectional study was conducted in the Department of Periodontology and Oral Implantology after receiving ethical approval from the Institutional Review Committee-Kathmandu University School of Medical Sciences (IRC-KUSMS Reference Number: 8/24). Written informed consent was taken from all the participants before starting the research procedure. Patients with a diagnosed case of periodontitis (interdental CAL is detectable at ≥2 nonadjacent teeth, or buccal or oral CAL ≥3 mm with pocketing ≥3 mm is detectable at ≥2 teeth) were included in the study according to the 2017 World Workshop on the Classification of Periodontal and Peri-Implant Diseases and Conditions. For sample size, an extensive literature search was done, which suggested five to 10 participants per question were sufficient for the instrument's validation [14]. Thus, taking into consideration six participants per question, the final sample size was 85. Informed written consent was taken before demographic data collection. All the participants were requested to fill up the questionnaire by themselves. About 5–10 min were given to fill up the 14 questions on one page. The questionnaire was adapted and translated following the guidelines provided by Beaton et al. [15]. The process involves several steps, including “initial translation, synthesis of the translation, back translation, expert committee review, testing of the prefinal version, and finalizing the version.”

### 2.2. Initial Translation

The original OHIP-14-PD in English was translated into Nepali with the assistance of bilingual native speakers. For this, two translators (first T1: one dental clinician, second T2: English language teacher from the university, who do not know about the concepts, purpose, or basis of the translation process) were instructed properly about the wording and the sequence of the questions. Both translators were good at both the native as well as in the English language. They were asked to translate with simple wording so that native language speaker participants could easily understand and fill up the form.

### 2.3. Synthesis of the Translation

Translated questionnaires (T1, T2) along with written reports from both translators were received. The principal investigator was involved in comparing and contrasting both questionnaires; differences in opinion and wording were resolved through proper discussion, and a single common translated version (T-12) was then prepared.

### 2.4. Back Translation

To prevent the discrepancies between the T-12 and the original version of the questionnaire, back translation was planned with the help of two other translators (one faculty from oral medicine and radiology, BT1, and one faculty from community dentistry, BT2). Both of them were proficient in both languages and unaware of the original version of the questionnaire. They were requested to submit a written report detailing the back-translation procedure.

### 2.5. Expert Committee

An expert committee was formed with the help of two expert periodontists, all the translators, and a few senior faculty members from various dental departments. Then, all the translated versions were compared. Members raised a concern about some wording that may be difficult to understand by the participants. Those words were either modified into simpler forms or removed by the expert committee. Further discrepancies among the committee members were resolved by discussion, and a prefinal version of the Nepali OHIP-14-PD was created.

### 2.6. Test of the Prefinal Version

The prefinal version was introduced among the randomly selected sample of 34 participants with chronic PD to ensure the quality of the questionnaire (content validity). They were allowed to fill up the questionnaire by themselves on a “five-point Likert scale (0 = never, 1 = hardly ever, 2 = occasionally, 3 = fairly often, 4 = very often).” After that, participants were interviewed about their level of understanding and difficulties, and the final version of the OHIP-14-PD Nepali language version was prepared.

The final version of the Nepalese version of OHIP-14-PD was then introduced among 85 patients diagnosed with PD to find out the internal consistency of the questionnaire. A random sample of 34 patients was allowed to fill up the questions in a 1-week interval and was asked to respond on a 5-point Likert scale to determine the test–retest reliability.

### 2.7. Statistical Analysis

Data obtained were first entered into the latest version of Microsoft Excel, and further analysis was done using Statistical Package for Social Sciences (SPSS) version 21. Descriptive data were presented as mean, frequency, and standard deviation. The internal consistency of the questionnaire was calculated using Cronbach's alpha value. Spearman's correlation coefficient was used to determine the validity. Cohen's kappa values were used to find out the agreement among the responses in the test–retest process. The intraclass correlation coefficient (ICC) was used to find out the consistency of the agreement with a 95% confidence interval.

## 3. Results

All the processes were carried out carefully, which resulted in similar forward and backward translation. Minor discrepancies were solved by discussion among the members of the expert committee. The demographic data of the study participants are presented in Table 1.

### 3.1. Reliability

The internal consistency was good for the translated and validated questionnaire to use in the Nepali hospital setup to evaluate the impact of chronic PD on OHRQoL (Cronbach's alpha value = 0.868, range = 0.85–0.86) (Table 2). The pre- and post-test Cohen's kappa value showed good consistency of agreement. Though the Cohen's kappa values were low initially due to the self-administered questionnaire, after a specified time, those participants were again interviewed, and then the Cohen's kappa value increased significantly (Table 3).

**Figure 1:**
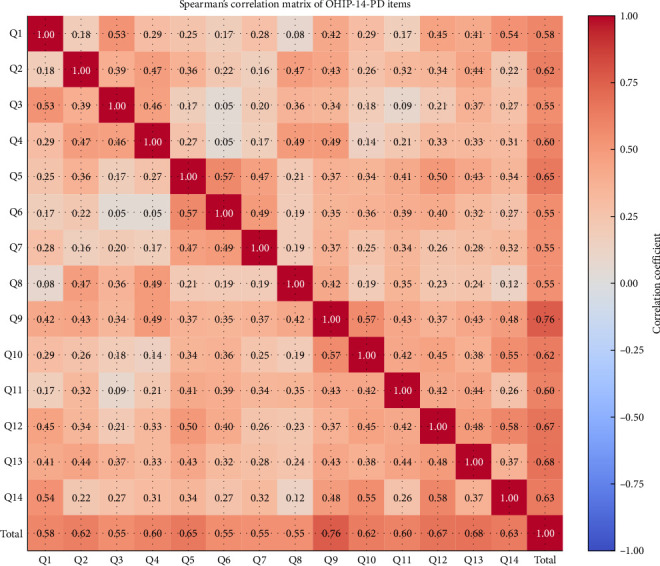
Representation of Spearman's correlation coefficient among all 14 items of the questionnaire.

The validity of the questionnaire was determined using Spearman's correlation coefficient. Spearman's correlation coefficient for the total OHIP-14-PD Nepali version was 0.585, which is greater than the table value, that is, significant (0.212) (Figure 1). The intraclass coefficient for the total OHIP-14-PD Nepali version was 0.868 (95% CI = 0.823–0.906).

## 4. Discussion

QoL of individuals is defined as “perceptions of their position in life in the context of culture and value systems in which they live, and in relation to their goals, expectations, standards, and concerns” [16]. Researchers have focused on the role of oral health in QoL and the association between various parameters like oral health, mental and social well-being, and functions [17–19].

The World Health Organization (WHO) views OHRQoL as a crucial component of the Global Oral Health Program [20] as health is considered a multi-dimensional phenomenon. Since then, there have been numerous scales to measure the OHRQoL in various oral diseases and conditions. Initially, there was a long version of OHIP-49, which was later shortened to make it easier to apply in clinical research settings (OHIP-14).

OHIP-14 has been used to find out the association between QoL and various oral health conditions by various authors [21–25]. PD is a chronic inflammatory condition that has a negative impact on OHRQoL. Initially, several authors used either OHIP-49 or OHIP-14 to find out the effects of chronic PD on OHRQoL [6, 26–29]. Afterwards, researchers focused on the effects of nonsurgical periodontal therapy [30–32] or surgical periodontal therapy [33–36] on OHRQoL of the patients with chronic PDperiodontal disease.

OHIP-14-PD was initially developed by Rodriguez and Moral [12] to overcome the problems that arose due to the lack of scale to evaluate the effect of chronic PD in OHRQoL in individuals with chronic PD. It includes seven dimensions of QoL: “functional limitation, physical pain, psychological discomfort, physical disability, psychological disability, social disability, and handicap” as in the original OHIP-14. They created a new instrument, as OHIP-14 to measure the effects of chronic PD on OHRQoL and validated it in the Mexican population.

PD is common among Nepalese people and is a leading cause of premature tooth loss. Recognizing the clinical significance of chronic PD in Nepal and the absence of a suitable tool to measure its impact on OHRQoL, we decided to validate and adapt the Mexican/English version of the OHIP-14-PD questionnaire into Nepali.

For the validation and cross-cultural adaptation of OHIP-14-PD, the guidelines given by Beaten et al. [15] were followed. The steps are included in sequential order: “initial translation, synthesis of the translations, back translation, expert committee, test of prefinal version, final version.” All these steps were followed carefully to develop the final Nepalese version of the OHIP-14-PD. Problems arose during the adaptation of the English word into the Nepalese language. Direct conversion was not legible to the participants. So, some terminologies according to the Nepalese clinical scenario were modified to enable the understandability of the questionnaire by the participants.

In our study, Cohen's kappa values improved notably after providing clarification to participants. These findings highlight that while the translated items were generally understood, some respondents initially faced difficulty interpreting certain terms. Such challenges are not uncommon in cross-cultural adaptation of instruments, as differences in language, literacy levels, and cultural context may influence comprehension. The improvement after clarification indicates that the content itself was relevant and acceptable, but subtle wording issues may have limited initial understanding. This underlines the importance of continuous refinement of translated tools to ensure clarity and minimize misinterpretation.

Internal consistency of the questionnaire was assessed with Cronbach's coefficient alpha. All the questions showed the α-values greater than 0.85 and an average value of 0.868, which is slightly lower than that of the Mexican version [37]. The higher reliability value in this case could be attributed to the smaller sample size in comparison to our study. However, the overall *α*-value in the present study was good (>80) according to the interpretation criteria. The corrected item-total values were greater than 0.2 (recommended value). From these findings, we can conclude that all the questions demonstrate good reliability and internal consistency.

The test–retest reliability was conducted in the interval of 1 week, which is long enough to avoid memory-based replies to the questionnaire [38]. Initially, there was very minimal agreement in Q4, moderately weak agreement in Q5, Q7, Q8, Q9, Q10, Q13, and Q14, and moderate agreement in Q1, Q2, Q3, Q6, and Q12. The reasons behind this may be due to a lack of knowledge about the nature of the study, random filling of the questionnaire, participants being in a rush, confusion regarding certain terminologies, etc.

Participants were once again assembled, briefed about the study's nature and significance, and requested to complete the questionnaire with adequate time and a certain level of seriousness. The same process of retesting was repeated in a 1-week interval. The results showed a dramatic change in Cohen's kappa value: Q1, Q8, Q10, Q13, Q14—strong agreement, Q2, Q6, Q11—almost perfect, and Q3, Q4, Q5—moderate agreement. These results indicate the need for a thorough explanation to the participants before providing the questionnaire form. The findings of the present study were slightly different from the original study in the Mexican population, where Q1−6, Q8−14 showed perfect agreement and Q7 showed very high agreement [12].

Content validation was done with the help of an expert panel. Construct validity means whether the questions measure what is intended to be measured and were measured with the help of Spearman's correlation coefficient and the ICC. Results from both tests showed validation of the current set/Nepali-language version of the questionnaire.

## 5. Conclusions

The Nepali language version of OHIP-14-PD is reliable. The content/construct-validated tool can be used to measure the effects of chronic PD on OHRQoL.

## 6. Limitations of the Study

This study has several limitations that should be considered. First, cultural factors may have influenced participants' interpretation of certain items, which could affect the applicability of the tool across different regions or subgroups within Nepal. Second, the study was conducted in a single tertiary care hospital, and therefore, the findings may not be fully generalizable to the broader Nepalese population. Third, as the OHIP-14-PD is a self-administered questionnaire, there is a potential for response bias, including social desirability bias or misunderstanding of certain terms. This may explain the initially lower Cohen's kappa values, which improved after clarification. Finally, while the translation and adaptation followed rigorous international guidelines, subtle differences in language comprehension and literacy levels across populations may still influence responses. These limitations suggest that further research is needed to test the Nepalese OHIP-14-PD in diverse settings and populations to ensure its reliability and validity.

## Figures and Tables

**Table 1:** Representation of demographic parameters.

SN	Particulars	Frequency, *n* (%)
1	Age	—
	Average age	35.62 ± 13.56 years
	Less than 20	12 (14.1)
	21–30	24 (28.2)
	31–40	23 (27.1)
	41–50	12(14.1)
	51–60	10 (11.8)
	More than 60	4 (4.7)

2	Gender	—
	Male	47 (55.3)
	Female	37 (43.3)

**Table 2:** Representation of inter-item total statistics.

Item-total statistics						
	Mean ± standard deviation	Scale mean if item deleted	Scale variance if item deleted	Corrected item-total correlation	Squared multiple correlation	Cronbach's Alpha if item deleted
Q1	1.35 ± 1.351	14.41	95.197	0.490	0.532	0.862
Q2	0.991 ± 0.314	14.78	94.509	0.537	0.430	0.859
Q3	1.59 ± 1.330	14.18	96.266	0.456	0.484	0.863
Q4	1.99 ± 1.384	13.78	94.557	0.500	0.494	0.861
Q5	0.76 ± 1.161	15.00	95.524	0.576	0.495	0.857
Q6	1.15 ± 1.286	14.61	96.669	0.459	0.467	0.863
Q7	1.19 ± 1.160	14.58	98.009	0.461	0.373	0.863
Q8	1.46 ± 1.332	14.31	96.477	0.447	0.421	0.864
Q9	1.79 ± 1.337	13.98	90.333	0.701	0.593	0.849
Q10	1.01 ± 1.401	14.75	93.545	0.532	0.516	0.859
Q11	0.89 ± 1.205	14.87	96.138	0.523	0.410	0.860
Q12	0.27 ± 0.746	15.49	100.086	0.623	0.555	0.859
Q13	0.71 ± 1.078	15.06	95.818	0.614	0.439	0.856
Q14	0.61 ± 1.013	15.15	97.512	0.570	0.551	0.858

**Table 3:** Representation of test–retest reliability using Cohen's kappa value.

SN	Cohen's Kappa value initially by self-administered questionnaire	Cohen's Kappa value after interviewing
Q1	0.694	0.871
Q2	0.746	0.958
Q3	0.743	0.788
Q4	0.346	0.799
Q5	0.589	0.792
Q6	0.738	0.915
Q7	0.581	0.910
Q8	0.525	0.850
Q9	0.580	0.809
Q10	0.405	0.816
Q11	0.663	0.903
Q12	0.751	0.878
Q13	0.566	0.873
Q14	0.574	0.897

## Data Availability

The data that support the findings of this study are available from the corresponding author upon reasonable request. The requests may be directed to the following email address: drarjunrijal@kusms.edu.np.

## Nomenclature

ICC: Intraclass coefficient,
IRC: Institutional Review Committee,
OHIP-14-PD: Oral health impact profile-14-periodontal disease.

## Supporting Information

Supporting Information Representation of Spearman's correlation coefficient among all 14 items of the questionnaire. (Represented as a figure in the manuscript).
