# Supplementary material for: Evaluation of a Nepalese-Language Version of the Oral Health Impact Profile Scale Applied to Periodontal Disease (OHIP-14-PD): A Reliability and Validation Study
Source: Int J Dent. 2025 Oct 14;2025:7031395. doi: 10.1155/ijod/7031395 (PMC12539986; doi:10.1155/ijod/7031395)
Supplement: Supporting Information — Representation of Spearman's correlation coefficient among all 14 items of the questionnaire. (Represented as a figure in the manuscript). [file 7031395.f1.docx]

**Representation of Spearman’s correlation coefficient among all 14 items of the questionnaire. (Represented as a figure in the manuscript)**

| **Correlations** | | | | | | | | | | | | | | | | |
| --- | --- | --- | --- | --- | --- | --- | --- | --- | --- | --- | --- | --- | --- | --- | --- | --- |
|  | | Q1 | Q2 | Q3 | Q4 | Q5 | Q6 | Q7 | Q8 | Q9 | Q10 | Q11 | Q12 | Q13 | Q14 | Total |
| Q1 | Pearson Correlation | 1 | .183 | .526^**^ | .295^**^ | .251^*^ | .174 | .276^*^ | .081 | .417^**^ | .293^**^ | .169 | .447^**^ | .407^**^ | .536^**^ | .585^**^ |
|  | Sig. (2-tailed) |  | .093 | .000 | .006 | .021 | .111 | .011 | .462 | .000 | .006 | .121 | .000 | .000 | .000 | .000 |
|  | N | 85 | 85 | 85 | 85 | 85 | 85 | 85 | 85 | 85 | 85 | 85 | 85 | 85 | 85 | 85 |
| Q2 | Pearson Correlation | .183 | 1 | .386^**^ | .471^**^ | .365^**^ | .219^*^ | .158 | .472^**^ | .432^**^ | .259^*^ | .322^**^ | .343^**^ | .443^**^ | .220^*^ | .623^**^ |
|  | Sig. (2-tailed) | .093 |  | .000 | .000 | .001 | .044 | .149 | .000 | .000 | .017 | .003 | .001 | .000 | .043 | .000 |
|  | N | 85 | 85 | 85 | 85 | 85 | 85 | 85 | 85 | 85 | 85 | 85 | 85 | 85 | 85 | 85 |
| Q3 | Pearson Correlation | .526^**^ | .386^**^ | 1 | .463^**^ | .175 | .051 | .197 | .356^**^ | .339^**^ | .181 | .091 | .210 | .371^**^ | .269^*^ | .554^**^ |
|  | Sig. (2-tailed) | .000 | .000 |  | .000 | .108 | .642 | .070 | .001 | .002 | .097 | .406 | .054 | .000 | .013 | .000 |
|  | N | 85 | 85 | 85 | 85 | 85 | 85 | 85 | 85 | 85 | 85 | 85 | 85 | 85 | 85 | 85 |
| Q4 | Pearson Correlation | .295^**^ | .471^**^ | .463^**^ | 1 | .265^*^ | .054 | .165 | .487^**^ | .487^**^ | .141 | .213 | .326^**^ | .325^**^ | .311^**^ | .596^**^ |
|  | Sig. (2-tailed) | .006 | .000 | .000 |  | .014 | .620 | .132 | .000 | .000 | .197 | .050 | .002 | .002 | .004 | .000 |
|  | N | 85 | 85 | 85 | 85 | 85 | 85 | 85 | 85 | 85 | 85 | 85 | 85 | 85 | 85 | 85 |
| Q5 | Pearson Correlation | .251^*^ | .365^**^ | .175 | .265^*^ | 1 | .574^**^ | .466^**^ | .209 | .374^**^ | .345^**^ | .407^**^ | .500^**^ | .429^**^ | .336^**^ | .647^**^ |
|  | Sig. (2-tailed) | .021 | .001 | .108 | .014 |  | .000 | .000 | .055 | .000 | .001 | .000 | .000 | .000 | .002 | .000 |
|  | N | 85 | 85 | 85 | 85 | 85 | 85 | 85 | 85 | 85 | 85 | 85 | 85 | 85 | 85 | 85 |
| Q6 | Pearson Correlation | .174 | .219^*^ | .051 | .054 | .574^**^ | 1 | .491^**^ | .188 | .351^**^ | .362^**^ | .394^**^ | .403^**^ | .316^**^ | .265^*^ | .553^**^ |
|  | Sig. (2-tailed) | .111 | .044 | .642 | .620 | .000 |  | .000 | .085 | .001 | .001 | .000 | .000 | .003 | .014 | .000 |
|  | N | 85 | 85 | 85 | 85 | 85 | 85 | 85 | 85 | 85 | 85 | 85 | 85 | 85 | 85 | 85 |
| Q7 | Pearson Correlation | .276^*^ | .158 | .197 | .165 | .466^**^ | .491^**^ | 1 | .190 | .371^**^ | .248^*^ | .338^**^ | .257^*^ | .283^**^ | .316^**^ | .546^**^ |
|  | Sig. (2-tailed) | .011 | .149 | .070 | .132 | .000 | .000 |  | .082 | .000 | .022 | .002 | .018 | .009 | .003 | .000 |
|  | N | 85 | 85 | 85 | 85 | 85 | 85 | 85 | 85 | 85 | 85 | 85 | 85 | 85 | 85 | 85 |
| Q8 | Pearson Correlation | .081 | .472^**^ | .356^**^ | .487^**^ | .209 | .188 | .190 | 1 | .416^**^ | .188 | .349^**^ | .233^*^ | .244^*^ | .125 | .546^**^ |
|  | Sig. (2-tailed) | .462 | .000 | .001 | .000 | .055 | .085 | .082 |  | .000 | .084 | .001 | .032 | .024 | .255 | .000 |
|  | N | 85 | 85 | 85 | 85 | 85 | 85 | 85 | 85 | 85 | 85 | 85 | 85 | 85 | 85 | 85 |
| Q9 | Pearson Correlation | .417^**^ | .432^**^ | .339^**^ | .487^**^ | .374^**^ | .351^**^ | .371^**^ | .416^**^ | 1 | .567^**^ | .429^**^ | .368^**^ | .427^**^ | .484^**^ | .763^**^ |
|  | Sig. (2-tailed) | .000 | .000 | .002 | .000 | .000 | .001 | .000 | .000 |  | .000 | .000 | .001 | .000 | .000 | .000 |
|  | N | 85 | 85 | 85 | 85 | 85 | 85 | 85 | 85 | 85 | 85 | 85 | 85 | 85 | 85 | 85 |
| Q10 | Pearson Correlation | .293^**^ | .259^*^ | .181 | .141 | .345^**^ | .362^**^ | .248^*^ | .188 | .567^**^ | 1 | .424^**^ | .452^**^ | .380^**^ | .548^**^ | .625^**^ |
|  | Sig. (2-tailed) | .006 | .017 | .097 | .197 | .001 | .001 | .022 | .084 | .000 |  | .000 | .000 | .000 | .000 | .000 |
|  | N | 85 | 85 | 85 | 85 | 85 | 85 | 85 | 85 | 85 | 85 | 85 | 85 | 85 | 85 | 85 |
| Q11 | Pearson Correlation | .169 | .322^**^ | .091 | .213 | .407^**^ | .394^**^ | .338^**^ | .349^**^ | .429^**^ | .424^**^ | 1 | .416^**^ | .443^**^ | .258^*^ | .604^**^ |
|  | Sig. (2-tailed) | .121 | .003 | .406 | .050 | .000 | .000 | .002 | .001 | .000 | .000 |  | .000 | .000 | .017 | .000 |
|  | N | 85 | 85 | 85 | 85 | 85 | 85 | 85 | 85 | 85 | 85 | 85 | 85 | 85 | 85 | 85 |
| Q12 | Pearson Correlation | .447^**^ | .343^**^ | .210 | .326^**^ | .500^**^ | .403^**^ | .257^*^ | .233^*^ | .368^**^ | .452^**^ | .416^**^ | 1 | .485^**^ | .582^**^ | .666^**^ |
|  | Sig. (2-tailed) | .000 | .001 | .054 | .002 | .000 | .000 | .018 | .032 | .001 | .000 | .000 |  | .000 | .000 | .000 |
|  | N | 85 | 85 | 85 | 85 | 85 | 85 | 85 | 85 | 85 | 85 | 85 | 85 | 85 | 85 | 85 |
| Q13 | Pearson Correlation | .407^**^ | .443^**^ | .371^**^ | .325^**^ | .429^**^ | .316^**^ | .283^**^ | .244^*^ | .427^**^ | .380^**^ | .443^**^ | .485^**^ | 1 | .374^**^ | .676^**^ |
|  | Sig. (2-tailed) | .000 | .000 | .000 | .002 | .000 | .003 | .009 | .024 | .000 | .000 | .000 | .000 |  | .000 | .000 |
|  | N | 85 | 85 | 85 | 85 | 85 | 85 | 85 | 85 | 85 | 85 | 85 | 85 | 85 | 85 | 85 |
| Q14 | Pearson Correlation | .536^**^ | .220^*^ | .269^*^ | .311^**^ | .336^**^ | .265^*^ | .316^**^ | .125 | .484^**^ | .548^**^ | .258^*^ | .582^**^ | .374^**^ | 1 | .634^**^ |
|  | Sig. (2-tailed) | .000 | .043 | .013 | .004 | .002 | .014 | .003 | .255 | .000 | .000 | .017 | .000 | .000 |  | .000 |
|  | N | 85 | 85 | 85 | 85 | 85 | 85 | 85 | 85 | 85 | 85 | 85 | 85 | 85 | 85 | 85 |
| Total | Pearson Correlation | .585^**^ | .623^**^ | .554^**^ | .596^**^ | .647^**^ | .553^**^ | .546^**^ | .546^**^ | .763^**^ | .625^**^ | .604^**^ | .666^**^ | .676^**^ | .634^**^ | 1 |
|  | Sig. (2-tailed) | .000 | .000 | .000 | .000 | .000 | .000 | .000 | .000 | .000 | .000 | .000 | .000 | .000 | .000 |  |
|  | N | 85 | 85 | 85 | 85 | 85 | 85 | 85 | 85 | 85 | 85 | 85 | 85 | 85 | 85 | 85 |
| **. Correlation is significant at the 0.01 level (2-tailed). | | | | | | | | | | | | | | | | |
| *. Correlation is significant at the 0.05 level (2-tailed). | | | | | | | | | | | | | | | | |
